# Estrogen Receptor Signaling Pathways Involved in Invasion and Colony Formation of Androgen-Independent Prostate Cancer Cells PC-3

**DOI:** 10.3390/ijms22031153

**Published:** 2021-01-25

**Authors:** Ana Paola G. Lombardi, Renan P. Cavalheiro, Catarina S. Porto, Carolina M. Vicente

**Affiliations:** 1 Laboratory of Experimental Endocrinology, Department of Pharmacology, Escola Paulista de Medicina, Universidade Federal de São Paulo, Rua Pedro de Toledo 669, Vila Clementino, São Paulo, SP 04039-032, Brazil; hermagroner@hotmail.com (A.P.G.L.); cmvicente.farmaco@gmail.com (C.M.V.); 2 Department of Biochemistry, Escola Paulista de Medicina, Universidade Federal de São Paulo, São Paulo, SP 04039-032, Brazil; rpcavalheiro@gmail.com

**Keywords:** estrogen receptor, SRC, PI3K/AKT, β-catenin, PC-3 cells

## Abstract

Castration-resistant prostate cancer (CRPC) is an advanced and androgen-independent form of prostate cancer. Recent studies of rapid actions mediated by estrogen in the prostate and its relationship with CRPC are emerging. We have previously shown that estrogen receptor (ER) promotes migration and invasion of the androgen-independent prostate cancer cells PC-3, but the signaling pathways involved in these events remain to be elucidated. Therefore, this study aimed to analyze the role of ERα and ERβ in the activation of SRC, and the involvement of SRC and PI3K/AKT on invasion and colony formation of the PC-3 cells. Our results showed that the activation of ERα (using ERα-selective agonist PPT) and ERβ (using ERβ-selective agonist DPN) increased phosphorylation of SRC in PC-3 cells. In the presence of the selective inhibitor for SRC-family kinases PP2, the effects of DPN and PPT on transmigration and soft agar colony formation assays were decreased. Furthermore, SRC is involved in the expression of the non-phosphorylated β-catenin. Finally, using PI3K specific inhibitor Wortmannin and AKT inhibitor MK2206, we showed that PI3K/AKT are also required for invasion and colony formation of PC-3 cells simulated by ER. This study provides novel insights into molecular mechanisms of ER in PC-3 cells by demonstrating that ER, located outside the cell nucleus, activates rapid responses molecules, including SRC and PI3K/AKT, which enhance the tumorigenic potential of prostate cancer cells, increasing cell proliferation, migration, invasion, and tumor formation.

## 1. Introduction

The androgen receptor (AR) is the classical target for prostate cancer treatment [1], and estrogens and their receptors have recently been implicated in prostate cancer development and tumor progression [2,3]. Initially, prostate cancer depends on androgen to evolve, but it can gradually progress to an androgen-independent form of the disease, also known as castration-resistant prostate cancer (CRPC) [2,4,5]. The molecular mechanisms involved in this stage of the disease are not fully understood and the current therapies are insufficient to improve the survival of patients.

Previous studies from our laboratory have already shown that, in androgen-independent prostate cancer cells PC-3 [6] and DU-145 [7], estrogen receptors (ER) ERα (ESR1) and ERβ (ESR2) are mostly located outside the nucleus of these cells, indicating the activation of rapid signaling pathways. In fact, the activation of these receptors increases the phosphorylation of ERK1/2 (extracellular signal-regulated kinase1 and 2) in both cell lines [6,7] and the phosphorylation of AKT (serine/threonine kinases) in PC-3 cells [8]. Furthermore, in PC-3 cells, the activation of ERβ decreases N-cadherin [9] and increases non-phosphorylated β-catenin levels [8,9]. The activation of ERβ promotes the increase of migration, invasion, and anchorage-independent growth of PC-3 cells through β-catenin pathway. The activation of ERα also plays a role on invasion and anchorage-independent growth of PC-3 cells [10]. However, the molecular mechanisms of the crosstalk between ER and β-catenin pathways in this cell remains to be elucidated.

It is important to mention that 17β-estradiol impacts normal and malignant tissue development via ERα and ERβ, either through ligand-activated transcriptional regulation (genomic pathway) or by triggering cytoplasmic-signaling cascades (nongenomic pathway). The possible convergence of genomic and nongenomic pathways on target genes is an attractive mechanism by which ER can finely regulate gene expression in different cells [11,12]. Indeed, evidence indicates that a pool of ER located in the cytoplasm and/or at the plasma membrane forms multiprotein complexes leading to the activation of downstream signaling molecules. ER may interact with SRC (non-receptor tyrosine kinase) out of the nucleus to activate extranuclear signaling pathways, such as ERK1/2 and AKT, in breast cancer cells [11]. In addition, the extranuclear complex of ERα:SRC:PLCγ (phospholipase Cγ) plays a role in activation of the tumor-protective anticipatory UPR (unfolded protein response) (UPR), thereby increasing the resilience of breast cancer cells [13]. SRC, which is induced by various cellular signal molecules and has a great effect in regulating numerous processes, including cell growth, differentiation, adhesion and the migration signaling pathway [14,15,16], is highly expressed in several prostate cancer cell lines [17,18,19]; as well as in most tissues obtained from prostate cancer [17,19,20]. Studies have also demonstrated that the phosphorylation of tyrosine 654 from β-catenin by SRC reduces the association of β-catenin with E-cadherin and α-catenin [21,22]. When SRC phosphorylates E-cadherin at residue Y860 and β-catenin at residue Y654, no interaction between E-cadherin/β-catenin occurs. β-catenin is then degraded or remain stabilized in the cytoplasm, and E-cadherin will be directed via Haikai for degradation [23]. Whether ER activates SRC and plays a role in regulating the expression and/or activity of β-catenin remains to be investigated.

SRC can combine with another non-receptor protein tyrosine kinase, FAK (focal adhesion kinase) to form a dual-kinase complex, which coordinate cell behavior through regulating downstream pathways and molecules, including AKT, p38 and ERK [24]. PI3K (phosphatidylinositol 3-kinase) and AKT are also involved in the development of prostate cancer and CRPC, but their functions are not yet fully elucidated [25]. The most common change in PI3K signaling in patients with advanced prostate cancer is the bi-allelic loss of tumor suppressor PTEN (phosphatase and tensin homolog) that occurs in 50% of patients. PTEN is the negative regulator of PI3K and its inactivation, by mutation or loss, results in the accumulation of phosphatidylinositol (3,4,5)-trisphosphate and phosphorylation of AKT [26]. The phosphorylation of AKT activates mTOR (mammalian target of rapamycin), which leads to cell division [27]. In vitro studies with prostate cancer cells have shown that PI3K/AKT/mTOR signaling is not only involved with proliferation [28] and apoptosis [29], but also with migration and invasion [30].

Therefore, this study aimed to examine the role of estrogen receptor in the activation of SRC, and the involvement of SRC and PI3K/AKT on invasion and colony formation of the androgen-independent prostate cancer cells PC-3.

## 2. Materials and Methods

### 2.1. Cell Culture

The human androgen-independent prostate cancer cell lines PC-3 (derived from bone metastasis) and DU-15 (derived from brain metastasis) were obtained from the American Type Culture Collection (Manassas, VA, United States). PC-3 and DU-145 cells were used in passages under 46 and 69, respectively, and cultures were carried out as previously described [6,8]. PC-3 and DU-145 cells were grown in RPMI 1640 medium without phenol red (GIBCO^®^, Rockville, MD, USA), supplemented with 10% of fetal bovine serum, HEPES (5.95 mg/mL) and gentamicin (0.02 mg/mL), in a humidified atmosphere with 5% CO_2_:95% air, at 37 °C, for 72 h. Then, the culture medium was replaced by serum free medium 24 h before the experiments. At this stage, the cells were 85–90% confluent, and the number of viable cells in each culture, as determined by trypan blue exclusion, was more than 90%. All experimental procedures were approved by the Research Ethical Committee at Escola Paulista de Medicina-Universidade Federal de São Paulo (#4330100615, 11 December 2015).

### 2.2. Western Blot Analysis for Detection of Total and Phosphorylated SRC and Non-Phosphorylated β-Catenin

PC-3 cells in culture medium without serum were incubated in the absence (control) and presence of 17β-estradiol (E2, 10 nM; Sigma Chemical Co., St.Louis, MO, USA) for 5, 15, 30 min and 1 and 2 h; ERβ-selective agonist DPN (10nM; 2,3-bis(4-hydroxyphenyl)-propionitrile, Tocris Bioscience, Bristol, UK) for 30 min and 1, 2, 4, and 24 h; ERα-selective agonist PPT (10 nM; 4,4′,4″-(4-propyl-(1H)-pyrazole-1,3,5-triyl)trisphenol, Tocris Bioscience) for 30 min and 1, 2, and 24 h. The cells were also untreated or pretreated with the selective inhibitor for SRC-family kinases PP2 (5 nM; 4-amino-3-(4-chlorophenyl)-1-(t-butyl)-1H-pyrazolo[3,4-d]pyrimidine, 4-amino-5-(4 chlorophenyl)-7-(t-butyl)pyrazolo[3,4-d]pyrimidine, Calbiochem, Darmstadt, Germany) for 30 min. Incubation was continued in the absence and presence of DPN (10 nM) for 30 min or PPT (10 nM) for 1 h. The cells were also untreated or pretreated with the ERα-selective antagonist MPP (10 nM; 1,3-bis(4-hydroxyphenyl)-4-methyl-5-[4-(2-piperidinylethoxy)phenol]-1H-pyrazole dihydrochloride, Tocris Bioscience) or with the ERβ-selective antagonist PHTPP (10 nM; 4-[2-phenyl-5,7-bis(trifluoromethyl)pyrazolo[1,5- a]pyrimidin-3-yl]phenol, Tocris Bioscience) for 30 min. Incubation was continued in the absence and presence of DPN (10 nM) for 30 min or PPT (10 nM) for 1 h. At these concentrations, the agonists and antagonists are highly selective, as previously reported [6,31,32,33]. Western blot analyses were performed as previously described [6,8], using rabbit monoclonal antibody raised against a synthetic phosphopeptide corresponding to Tyr419 of human SRC (Phosphorylated SRC #6943, Cell Signaling Technology, Boston, MA, USA, 1:1000 dilution) or antibody polyclonal raised against a synthetic peptide corresponding to human SRC (Total SRC, #2108, Cell Signaling Technology, 1:1000 dilution) or rabbit polyclonal antibody raised against a synthetic peptide corresponding to Ser 37 (Sr33/37/Thr41) of human non-phosphorylated β-catenin (#4270, Cell Signaling Technology, 1:600 dilution) or monoclonal rabbit antibody raised against a synthetic peptide corresponding to the amino-terminal of the human β-tubulin (#2128, Cell Signaling Technology, 1:2000 dilution) overnight at 4 °C. Apparent molecular masses were determined from molecular mass standards. Band intensities of phosphorylated SRC, total SRC, non-phosphorylated β-catenin, and β-tubulin from individual experiments were quantified by densitometric analysis of linear-range autoradiograms, using an Epson Expression 1680 scanner and the quick Scan 2000 WIN software (Helena Laboratories Co. Beaumont, TX, USA). Results were normalized based on expression of total SRC or β-tubulin in each sample and plotted (mean ± SEM) in relation to control (C = 1).

### 2.3. Protein Assays

Protein concentration was determined with the Bio-Rad protein assay, using bovine serum albumin as standard (Bio Rad Laboratories Inc., Hercules, CA, USA).

### 2.4. Cell Invasion Analysis

PC-3 or DU-145 cells (2 × 10^5^ cells) in serum free culture medium were seeded in ThincertR chambers (Greiner Bio-one, Kremsmünster, Austria) with polyethylene terephthalate membranes (8 mm pore size) pre-coated with 50 mL of phenol red-free Matrigel (1:10, BD, Corning). These chambers were placed in 24-well plates containing culture medium with 10% FBS in the lower chamber [10,34]. PC-3 or DU-145 cells in upper chambers were incubated in the absence (control) and presence of 17β-estradiol (E2, 10 nM) or DPN (10 nM) or PPT (10 nM) for 48 h at 37 °C. The cells were also untreated or pretreated with PP2 (5 nM) or PI3K specific inhibitor Wortmannin (1 µM; Sigma-Aldrich Co., St. Louis, MO, USA) or AKT inhibitor MK2206, [200 nM, 8-(4-(1-aminocyclobutyl)phenyl)]-9-phenyl-[1,2,4]triazolo[3,4f][1,6]naphthyridin-3(2H)-one, Selleck Chemicals, Kirby Drive, Houston, TX, USA) for 30 min. Incubation was continued in the absence and presence of E2 (10 nM) or DPN (10 nM) or PPT (10 nM) for 48 h at 37 °C. Cell invasion analyses were performed as previously described [10]. The chambers were washed thoroughly with 10 mM PBS, fixed in 4% paraformaldehyde for 30 min, and stained with 0.2% crystal violet for 10 min. Non-invading cells, from the membrane upper surface, were removed using a cotton swab. The membranes containing the invaded cells (under the surface of membrane), were photographed. Images of three random microscope fields were captured in duplicate, using an inverted optical microscope (Floid Cell Imaging Station, Life Technologies, Carlsbad, CA, USA). The areas of cell invasion were determined by Image J software. Results were plotted (mean ± SEM) in relation to control (C = 100) or agonists subtracted from the control (agonists = 100).

### 2.5. Colony Formation Analysis (Soft Agar)

PC-3 cells (6 × 10^3^ cells) in culture medium containing 10% FBS and 0.35% agarose (low melting, Sigma Chemical Co.) were seeded in 24-well plates pre-coated with 300 mL of 0.7% agarose at 4 °C for 30 min [10,34]. Cells were incubated at 37 °C for 2 h. Afterward, this culture medium was replaced by culture medium containing 10% SFB, pretreated with activated charcoal (0.25%) and dextran T-70 (0.0025%), for 24 h at 37 °C. PC-3 cells were incubated in the absence (control) and presence of E2 (10 nM), DPN (10 nM) or PPT (10 nM) for 3 weeks, with regular change in medium on every alternate day, at 37 °C. Cells were also untreated or pretreated with PP2 (5 nM), Wortmannin (1 µM) or MK2206 (200 nM), for 30 min. Incubation was continued in the absence and presence of E2 (10 nM) DPN (10 nM) or PPT (10 nM) for 3 weeks at 37 °C [10]. Colony formation analyses were performed as previously described [10]. Images of three random microscope fields, in duplicate, were captured using an inverted optical microscope (Floid Cell Imaging Station, Life Technologies). Image J software was used to determine the area of each colony. Only the spheroid-shaped colonies were considered for area calculation. Star- and spheroid-shaped colonies above 50 μm were counted using software Zen. Images are representative of three different experiments.

### 2.6. Immunofluorescence Analysis for the Detection of Non-Phosphorylated β-Catenin

PC-3 cells were grown as described above on coverslips coated with gelatin (0.1%, *w*/*v*) and placed into six-well plates. PC-3 cells in serum free culture medium were incubated in the absence (control) and presence of DPN (10 nM) or PPT (10 nM), for 2 h at 37 °C. The cells were also untreated or pretreated with PP2 (5 nM) for 30 min. Incubation was continued in the absence and presence of PPT (10 nM) or DPN (10 nM), for 2 h at 37 °C. The medium was removed. The cells were washed with PBS, fixed in 2% paraformaldehyde for 20 min at room temperature and washed with PBS containing 0.1 M glycine. Cells were then permeabilized with 0.01% saponin and blocked with PBS containing 1% bovine serum albumin (BSA) for 10 min at room temperature. The immunofluorescence analyses were performed as previously described [6,8] using a rabbit polyclonal antibody raised against a synthetic peptide corresponding to Ser 37 (Sr33/37/Thr41) of human non-phosphorylated β-catenin (#4270, Cell Signaling Technology) at 1:200 dilution and Alexa Fluor 488-labeled secondary antibody (anti rabbit antibody; Molecular Probes, Invitrogen, Carlsbad, CA, USA) at 1:300 dilution. Nuclear staining was performed with DAPI (40,6-diamidino-2-phenylindole, Sigma Chemical Co). Negative control was performed in the absence of the primary antibody. Images of five random microscope fields were captured, in duplicate, in each assay and analyzed using the software LAS-AF and Image J. Images are representative of at least three different experiments performed in duplicate.

### 2.7. Statistical Analysis

Data were expressed as mean ± SEM. Statistical analysis was carried out by ANOVA followed by the Newman–Keuls test for multiple comparisons or by Student *t*-test to compare the differences between two data. *p* values < 0.05 were accepted as significant.

## 3. Results

### 3.1. Activation of ERα and ERβ Increases the Phosphorylation of SRC in PC-3 Cells

**Figure 1:**
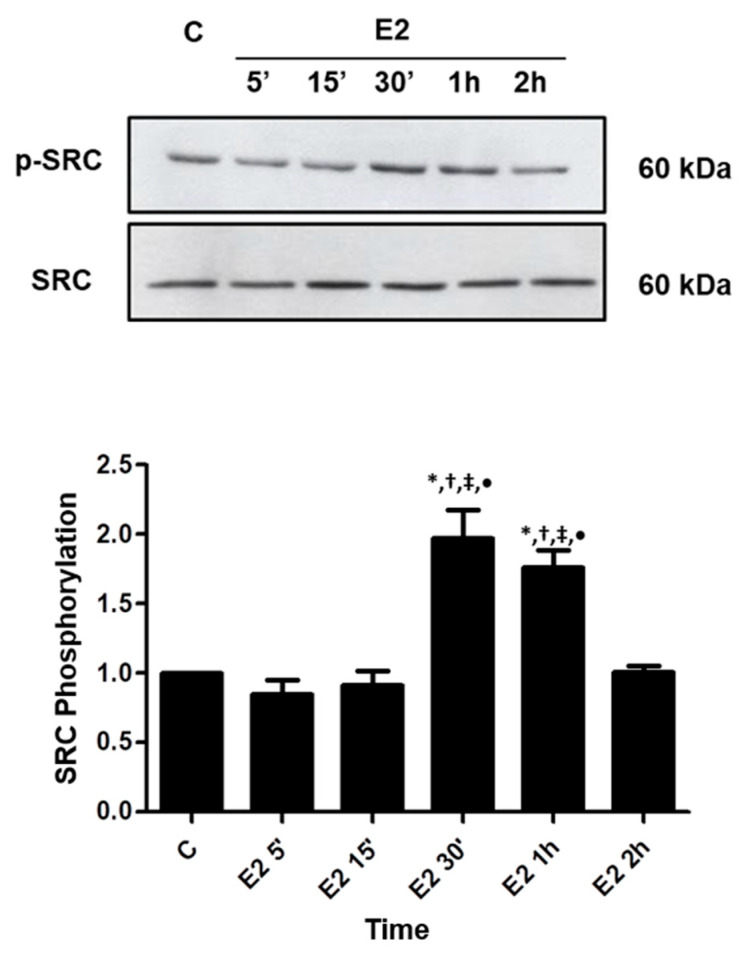
Effects of 17β-estradiol (E2) on SRC (Tyr419) phosphorylation in androgen-independent prostate cancer cells PC-3. The cells were incubated in the absence (C, control) and presence of E2 (10 nM) for 5, 15, 30 min, 1 and 2 h at 37 °C. Phosphorylated SRC (p-SRC) and total SRC were detected by Western Blot. The immunoassay was performed with anti-phosphorylated SRC (upper panel) and anti-total SRC antibodies (lower panel). The relative position of SRC was determined from the molecular weight standard. The data shown are representative of three independent experiments. Densitometric analysis was performed of the results obtained from each band, normalized by the expression of the total SRC and expressed in relation to the control (C = 1). Results were plotted (mean ± SEM) of three independent experiments. * Significantly different from the values obtained in relation to the control (C) (*p* < 0.05, ANOVA and Newman-Keuls). † Significantly different from E2 5 min (*p* < 0.05, ANOVA and Newman-Keuls). ‡ Significantly different from E2 15 min (*p* < 0.05, ANOVA and Newman-Keuls). • Significantly different from E2 2 h (*p* < 0.05, ANOVA and Newman-Keuls).

**Figure 2:**
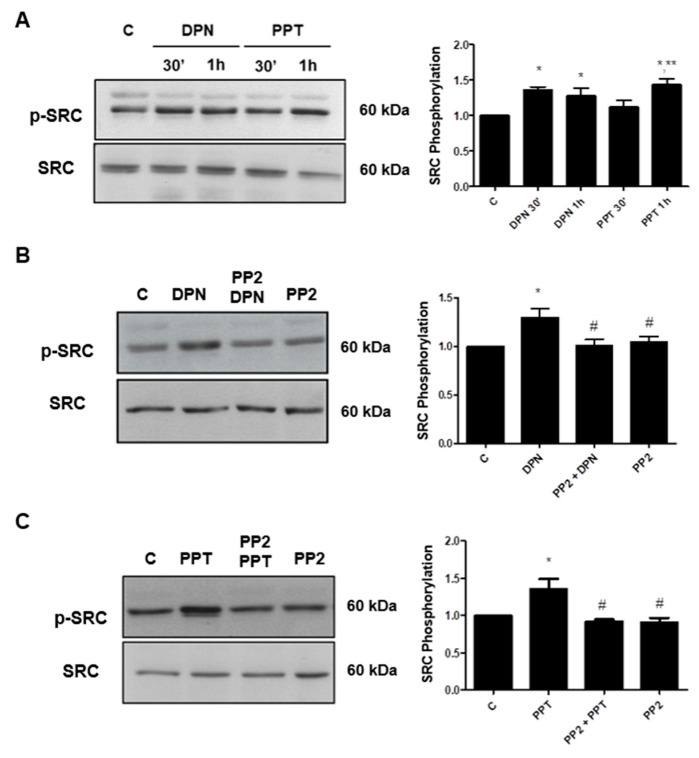
Effects of ERβ- (DPN) or ERα-selective agonists (PPT) on SRC (Tyr419) phosphorylation in androgen-independent prostate cancer cells PC-3. The cells were incubated in the absence (C, control) and presence of DPN or PPT (10 nM) for 30 min and 1 h at 37 °C (**A**). The cells were incubated in the absence (C, control) and presence of DPN (10 nM) for 30 min (**B**) or PPT (10 nM) (**C**) for 1 h. The cells were also pre-treated with the selective inhibitor for SRC-family kinases (PP2 5 nM, 30 min) and then incubated or not with DPN for 30 min (**B**) or PPT for 1 h (**C**). Phosphorylated SRC (p-SRC) and total SRC were detected by Western Blot. The immunoassay was performed with anti-phosphorylated SRC (upper panel) and total anti-SRC antibodies (lower panel). The relative position of SRC was determined from the molecular weight standard. The data shown are representative of four to six independent experiments. Densitometric analysis was performed of the results obtained from each band, normalized by the expression of the total SRC and expressed in relation to the control (C = 1). Results were plotted (mean ± SEM) of four to six independent experiments. * Significantly different from control (C) (*p* < 0.05, ANOVA and Newman-Keuls). ** Significantly different from PPT 30 min (*p* < 0.05, ANOVA and Newman-Keuls). # Significantly different from DPN for 30 min or PPT for 1 h (*p* < 0.05, ANOVA and Newman-Keuls).

17β-estradiol (E2) induced an increase in the phosphorylation of SRC (Tyr419) in PC-3 cells (Figure 1). The maximum increase (about 2-fold increase compared with control) was observed at 30 min after treatment. ERα-selective agonist PPT and ERβ-selective agonist DPN also increased the phosphorylation of SRC at 30 min and 1 h, respectively (Figure 2A).

In the presence of the selective inhibitor for SRC-family kinases (PP2, 5 nM), the effects of DPN and PPT on phosphorylation of SRC were blocked (Figure 2B,C).

**Figure 3:**
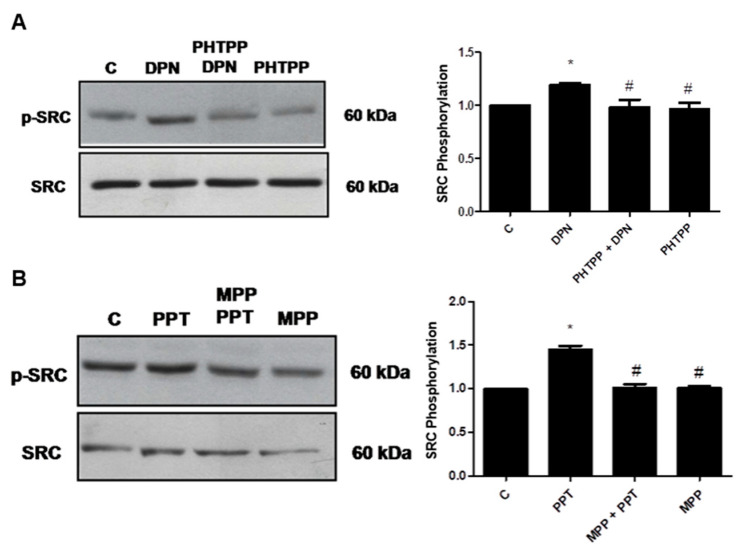
Effects of ERβ- (PHTTP) or ERα-selective antagonists (MPP) on SRC (Tyr419) expression and phosphorylation in androgen-independent prostate cancer cells PC-3 induced by DPN or PPT. The cells were incubated in the absence (C, control) and presence of DPN (10 nM) for 30 min (**A**) or PPT (10 nM) for 1 h (**B**) at 37 °C. The cells were also pre-treated with PHTPP (10 nM) (**A**) or MPP (10 nM) (**B**) for 30 min, and then incubated or not, respectively, with DPN for 30 min (**A**) or PPT for 1 h (**B**). Phosphorylated SRC (p-SRC) and total SRC were detected by Western Blot. The immunoassay was performed with anti-phosphorylated SRC (upper panel) and anti-total SRC antibodies (lower panel). The relative position of SRC was determined from the molecular weight standard. The data shown are representative of three independent experiments. Densitometric analysis was performed of the results obtained from each band, normalized by the expression of the total SRC and expressed in relation to the control (C = 1). Results were plotted (mean ± SEM) of three independent experiments. * Significantly different from control (C) (*p* < 0.05, ANOVA and Newman-Keuls). # Significantly different from DPN for 30 min or PPT for 1 h (*p* < 0.05, ANOVA and Newman-Keuls).

The involvement of each ER (ERα and ERβ) on phosphorylation of SRC was detected, using the respective selective antagonists MPP (10 nM) and PHTPP (10 nM). The effects of PPT and DPN on phosphorylation of SRC were blocked by their respective antagonists (Figure 3). It is important to mention that PP2, MPP and PHTPP were also incubated with the cells in the absence of the agonists, and the observed effects were similar to the control (Figure 2 and Figure 3).

### 3.2. SRC is Involved on Invasion and Colony Formation of PC-3 Cells Induced by ER Activation

**Figure 4:**
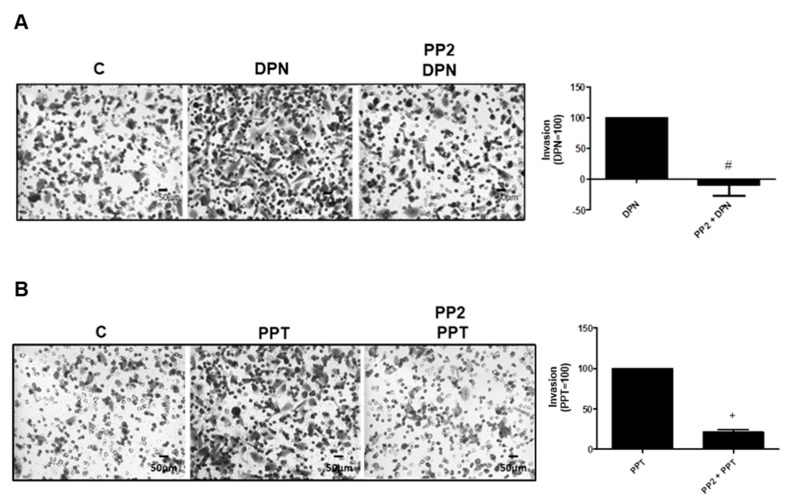
Effects of the selective inhibitor for SRC-family kinases (PP2) on the invasion of androgen-independent prostate cancer cells PC-3 induced by DPN and PPT. Cells were incubated in the absence (C, control) and in the presence of DPN (10 nM) (**A**) or PPT (10 nM) (**B**) for 48 h at 37 °C. The cells were pre-treated with PP2 (5 nM) for 30 min and then incubated or not with the DPN (**A**) or PPT (**B**) for 48 h. The membranes containing the invaded cells (under the surface of membrane), were photographed. Images of three random microscope fields, in duplicate, were captured using an inverted optical microscope. The areas of invaded cells were determined by Image J software. Results were plotted (mean ± SEM of three independent experiments) in relation to the DPN or PPT subtracted from the control (DPN = 100) (**A**) or (PPT = 100) (**B**). # Significantly different from DPN (*p* < 0.05, Student *t*-test). + Significantly different from PPT (*p* < 0.05, Student *t*-test). Images are representative of three different experiments.

To analyze the participation of SRC in the tumorigenicity of PC-3 cells, we performed invasion and colony formation assays. The treatment with ERβ-selective agonist DPN (10 nM) and ERα-selective agonist PPT (10 nM) for 48 h increased the invasion of PC-3 cells (Figure 4). In the presence of the selective inhibitor for SRC-family kinases PP2 (5 nM), the effects of DPN were blocked (100%) and PPT decreased 79%, suggesting the participation of SRC on the invasion of PC-3 cells (Figure 4). Cells were also incubated with PP2 in the absence of ER agonists, and the observed effects were similar to the control (data not shown).

The participation of SRC was also analyzed on invasion of DU-145 cells induced by activation of ER. The treatment with 17β-estradiol (E2 10 nM) for 48 h increased the invasion of DU-145 cells (Supplementary Figure S1). In the presence of the selective inhibitor for SRC-family kinases PP2 (5 nM), the effects of E2 were blocked (100%), suggesting the involvement of SRC also on the invasion of DU-145 cells (Supplementary Figure S1).

**Figure 5:**
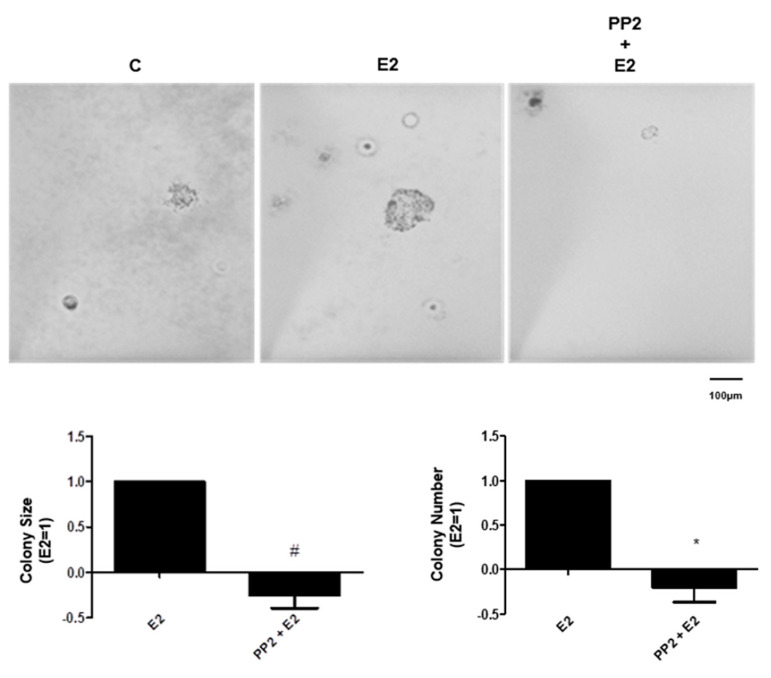
Effects of the selective inhibitor for SRC-family kinases (PP2) on size and number of the colony formed by androgen-independent prostate cancer cells PC-3 induced by 17β-estradiol (E2). Cells were incubated in the absence (C, control) and in the presence of 17β-estradiol (10 nM) for 3 weeks at 37 °C. The cells were also pre-treated with PP2 (5 nM) for 30 min and then incubated or not with E2. Representative image of PC-3 cell colony formation assays. The images were acquired, and the area of each colony was measured by the Image J software, and then the values obtained were expressed in relation to E2 and subtracted from the control (E2 = 1). The colonies were also counted with Zen software and the values of the number of colonies were expression in relation to E2 and subtracted from the control (E2 = 1). Results are expressed as (mean ± SEM) of three independent experiments. # Significantly different from E2 (*p* < 0.05, Student *t*-test) by #, * Significantly different from E2 (*p* < 0.05, Student *t*-test).

In the colony formation assay, treatment with E2 (10 nM) increased the size of the colonies when compared to the control, in the third week of treatment. The pretreatment with PP2 blocked the effect of E2 (Figure 5). The treatment with E2 also increased the number of colonies when compared to control, in the third week of treatment. The pretreatment with PP2 blocked this effect (Figure 5). Cells were also incubated with PP2 in the absence of ER agonists, and the observed effects were similar to the control (data not shown). These results suggest the involvement of SRC in the formation of colonies of PC-3 cells stimulated by E2.

### 3.3. The Increase in the Expression of the Non-Phosphorylated β-Catenin Induced by Activation of ERβ is Mediated by SRC

**Figure 6:**
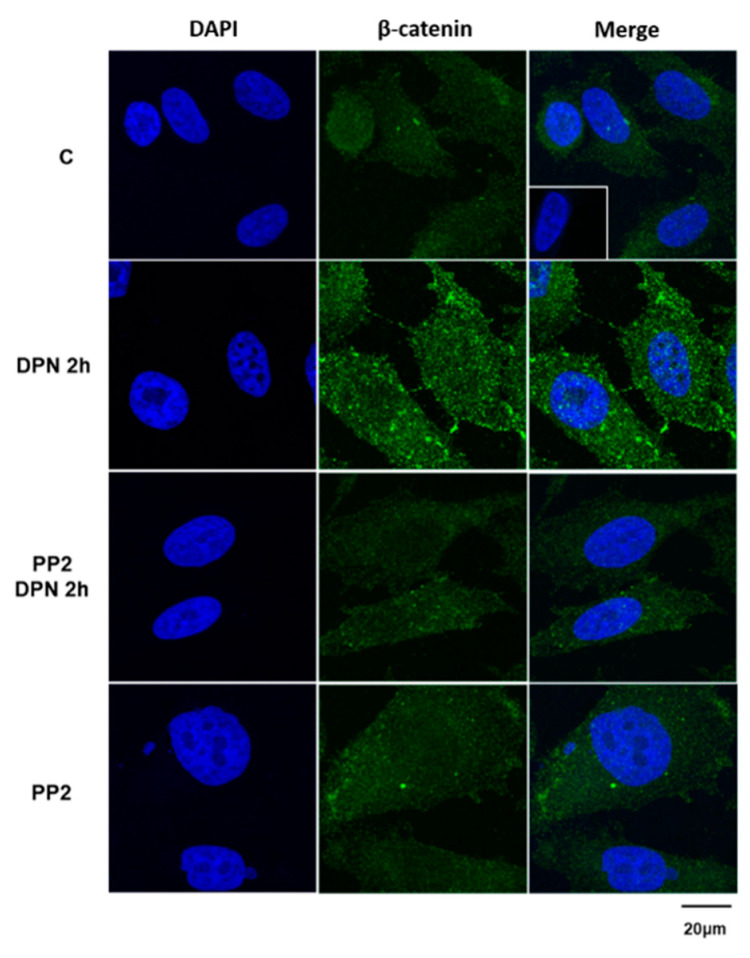
Effects of the selective inhibitor for SRC-family kinases (PP2) on the expression of non-phosphorylated β-catenin in androgen-independent prostate cancer cells PC-3 induced by DPN. Cells were incubated in the absence (C, control) and in the presence of DPN (10 nM) for 2 h at 37 °C. The cells were also pre-treated with PP2 (5 nM) for 30 min and then incubated or not with the DPN for 2 h. Positive immunostaining for non-phosphorylated β-catenin (green) was detected using the polyclonal antibody produced in rabbit by immunization with the synthetic peptide corresponding to the region around Serine 37 (Ser33/37/Thr41) of human β-catenin. Nuclei were stained with DAPI (blue). Negative control, in the absence of the primary antibody (detail). Results are representative of four independent experiments.

We previously demonstrated that ERs promote migration, invasion, and colony formation of PC-3 cells through β-catenin [10]. To analyze the participation of SRC on the expression of the non-phosphorylated β-catenin, we performed immunofluorescence analysis. The treatment with the ERβ-selective agonist DPN (10 nM) for 2 h increased the immunostaining of the non-phosphorylated β-catenin in the cytoplasm, near to the plasma membrane and cell nucleus, confirming our previous results [8] (Figure 6). This effect of DPN was blocked by the selective inhibitor for SRC-family kinases (PP2, 5 nM). In the presence of only PP2, the immunostaining of the β-catenin was similar to that detected in basal conditions (control, C). No immunostaining was detected in the negative control, performed in the absence of the primary antibody (insert).

To confirm the effect of the ERβ-selective agonist DPN on the expression of the non-phosphorylated β-catenin, Western blot analysis was also performed (Supplementary Figure S2). The activation of ERβ by DPN increased the expression of the non-phosphorylated β-catenin (Supplementary Figure S2). It is important to mention that the activation of ERα by PPT for 2 and 24 h did not alter the expression of the non-phosphorylated β-catenin. However, an increase in the expression of this protein was observed only after 48 h of incubation with PPT (data not shown).

### 3.4. PI3K/AKT are also Required for Invasion and Colony Formation of PC-3 Cells Simulated by ER

**Figure 7:**
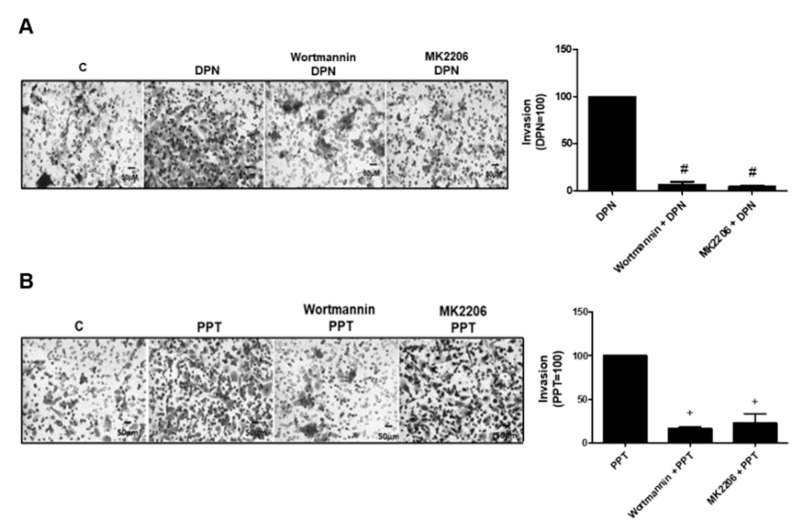
Effects of the PI3K specific inhibitor (Wortmannin) and AKT inhibitor (MK2206) on the invasion of androgen-independent prostate cancer cells PC-3 induced by DPN and PPT. Cells were incubated in the absence (C, control) and presence of DPN (10 nM) (**A**) or PPT (10 nM) (**B**) for 48 h at 37 °C. The cells were pre-treated with Wortmannin (1 µM) or MK2206 (200 nM) for 30 min, and then incubated or not with DPN (**A**) or PPT (**B**) for 48 h. Representative image of PC-3 cell invasion. The membranes containing the invaded cells (under the surface of membrane), were photographed. Images of three random microscope fields, in duplicate, were captured using an inverted optical microscope. The areas of invaded cells were determined by Image J software. Results were plotted (mean ± SEM) in relation to DPN or PPT and subtracted from the control (DPN = 100) (**A**) or PPT = 100) (**B**). # Significantly different from DPN (*p* < 0.05, ANOVA and Newman-Keuls). + Significantly different from PPT (*p* < 0.05, ANOVA and Newman-Keuls). Images are representative of three different experiments.

**Figure 8:**
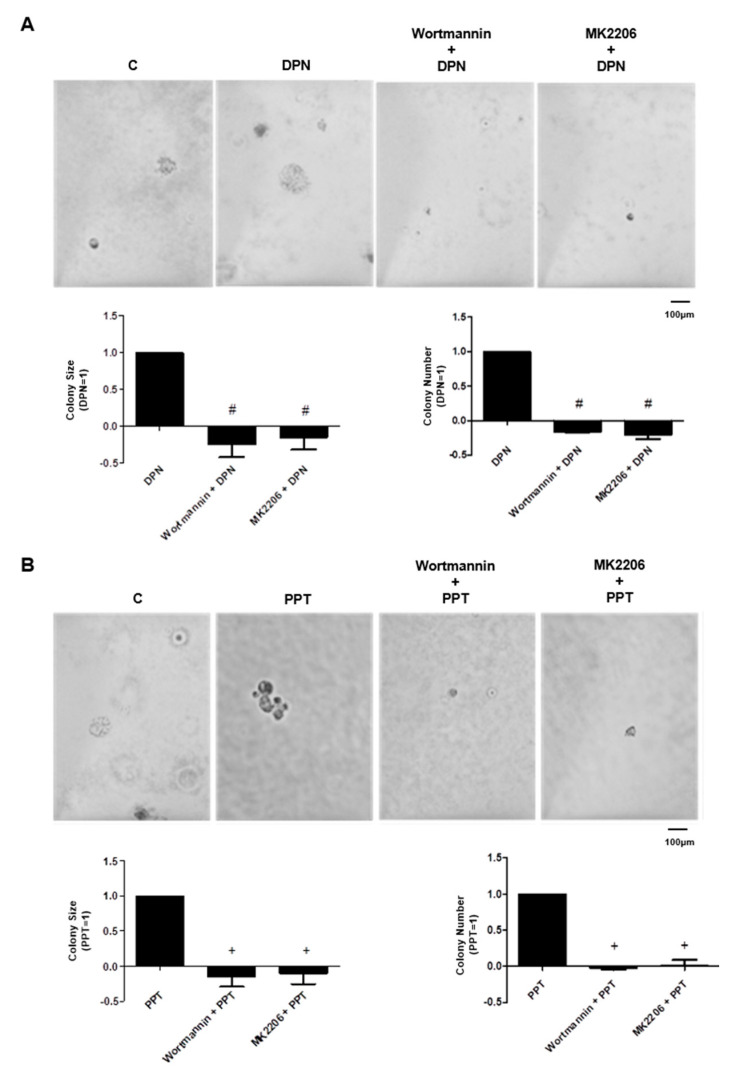
Effects of the PI3K specific inhibitors (Wortmannin) and AKT inhibitor (MK2206) on size and number of the colony formed by androgen-independent prostate cancer cells PC3 induced by DPN or PPT. Cells were incubated in the absence (C, control) and presence of DPN (10 nM) (**A**) and PPT (10 nM) (**B**) for 3 weeks at 37 °C. The cells were also pre-treated with Wortmannin (1 μM) or MK2206 (200 nM) for 30 min, and then incubated or not with DPN (**A**) or PPT (**B**). Representative image of PC-3 cell colony formation assays. The images were acquired, and the area of each colony was measured by the Image J software and the values obtained were expressed in relation to the DPN or PPT and subtracted from the control (DPN = 1) (**A**) or (PPT = 1) (**B**). The colonies were also counted with Zen software and the values of the number of colonies were expression in relation to DPN (**A**) or PPT (**B**) and subtracted from the control (DPN = 1) (**A**) or (PPT = 1) (**B**). Results are expressed as (mean ± SEM) of three independent experiments. # Significantly different from DPN (*p* < 0.05, ANOVA and Newman-Keuls). + Significantly different from PPT (*p* < 0.05, ANOVA and Newman-Keuls).

The treatment with ERβ-selective agonist DPN (10 nM) and ERα-selective agonist PPT (10 nM) for 48 h increased the invasion of PC-3 cells. In the presence of PI3K specific inhibitor (Wortmannin, 1 µM) and AKT inhibitor (MK2206, 200 nM), the effects of DPN were blocked (100%) by both inhibitors and the effects of PPT decreased by 80%, suggesting the participation of PI3K/AKT on the invasion of PC-3 cells stimulated by both agonists (Figure 7). Moreover, the pretreatment of PC-3 cells with Wortmannin (1 µM) and MK2206 (200 nM) blocked the effects of both agonists on size and number of the colonies (Figure 8). These results suggest the involvement of PI3K/AKT in the colony formation of PC-3 cells stimulated by DPN and PPT.

## 4. Discussion

Recent studies of rapid actions mediated by estrogen in the prostate and its relationship with the development of prostate cancer or with CRPC are emerging. Our laboratory showed that in androgen-independent prostate cancer PC-3 and DU-145 cells, the estrogen receptors ERα and ERβ are mostly located outside the cell nucleus [6,7]. The activation of ERα and ERβ can activate rapid cell signaling pathways in these cells, including an increase in the phosphorylation of ERK1/2 in PC-3 and DU-145 cells [6,7] and AKT in PC-3 cells [7,8], but not in DU-145 cells [7]. We now report that ER induces activation of SRC and PI3K/AKT, increases the expression of the non-phosphorylated β-catenin and enhances the invasion and colony formation of the PC-3 cells.

SRC and the non-receptor protein tyrosine kinases are downstream targets for cell surface receptors, and function as a link between the membrane receptors and the cytoplasmic signaling machinery, thereby regulating many fundamental cellular processes, including cell growth, differentiation, cell shape, migration and survival, and specialized cell signals [15]. All these processes, if deregulated, lead to tumor progression [35,36,37]. E2-ERα complex can enhances kinase activity by inducing binding of phosphotyrosine 537 of ERα to SH2 domain of SRC, changing the inactive conformation of SRC to active conformation [38]. In fact, in the present study, using the PC-3 cells, the activation of ER by E2, ERα- (PPT) or ERβ-selective agonists (DPN) leads to the phosphorylation of SRC (Tyr419). Furthermore, the selective inhibitor for SRC-family kinases (PP2) blocked the invasion of the PC-3 cells stimulated by DPN or PPT and also the invasion of the DU-145 cells stimulated by E2, indicating the involvement of ERβ-SRC and ERα-SRC on the invasion of both cells. In addition, activation of both ER by E2 increases the size and number of the colony formed by PC-3 cells [10] and present study. The pretreatment with the selective inhibitor for SRC-family kinases PP2 blunted these effects induced by E2, indicating the involvement of ER/SRC on tumor formation in vitro. The possible convergence of nongenomic and genomic pathways on target genes involved with invasion and colony formed by androgen-independent prostate cancer cells may be occurring.

It is important to mention that the activation of ERβ increases the expression of the non-phosphorylated β-catenin [8], and present data and ERβ-β-catenin-TCF/LEF complex is involved in proliferation of the PC-3 cells [8], migration, invasion, size and number of the colony formed by PC-3 cells [10]. In the present study, the pretreatment with the selective inhibitor for SRC-family kinases PP2 blocked the effect induced by DPN on expression of the non-phosphorylated β-catenin, indicating the involvement of SRC in the regulation of this protein. It is important to mention that the antibody used in the detection of the non-phosphorylated β-catenin is against the Ser33/37/Thr41 region, this region may be phosphorylated by GSK3β [39] and not by SRC.

SRC can phosphorylate β-catenin in Tyr654 [23]. Phosphorylation of β-catenin by members of the SRC family reduces the association of β-catenin with E-cadherin and α-catenin [21,22] increasing the levels of cytoplasmic β-catenin and translocation to the nucleus, where it interacts with the TCF/LEF transcription factor, resulting on the activation of target genes [40]. Whether ERβ-SRC complex also plays a role on the activation of β-catenin in Tyr654 in PC-3 cells remains to be determined. In the present study, we show that ERβ induces activation of SRC (30 min) (rapid action, nongenomic) and increases the levels of the expression of the non-phosphorylated β-catenin in the cytoplasm of PC-3 cells (2 h, genomic action).

Consistent with our results, study has shown that selective inhibitor for SRC-family kinases PP2 suppressed migration, invasion, and angiogenesis of PC3 and LNCAP cells via FAK [41]. Whether 17β-estradiol-ER-SRC plays a direct role or together with β-catenin and/or FAK on migration, invasion, and angiogenesis of PC3 cells remains to be explored.

Recently, new emerging roles for SRC have been described in the nuclear compartment. In the nucleus of normal and cancer cells, SRC is involved in several activities involving both its enzymatic activity as tyrosine kinase and its capability to interact with other protein thereby forming protein complexes. SRC participates in the regulation of chromatin reorganization and transcriptional activity of transcription factors, and it is surely involved in the oncogenic transformation of tumoral cells, by repressing some oncosuppressors [42]. The roles of SRC in the nuclear compartment of the prostate cancer cells remains to be explored.

Although several partners of extranuclear ER have been described in different cell types, the most conserved partners are SRC and PI3K [43]. The pathway, characterized by the formation of ER/SRC/PI3K and the subsequent activation of AKT, is present in normal breast tissue and is hyperactivated in aggressive breast tumors [44]. In PC-3 cells, our laboratory showed that E2 increases the phosphorylation of AKT [8]. In the present study was shown that the inhibitors of PI3K or AKT blocked the increase in cell invasion stimulated by DPN (100%) or PPT (80%), suggesting the involvement of ERβ-PI3K/AKT and ERα-PI3K/AKT on the invasion potential of PC-3 cells. In addition, the activation of ERβ by DPN or ERα by PPT increases the size and number of the colony formed by PC-3 cells [10 and present study). These effects were also blocked by inhibitors of PI3K or AKT. 

It is also important to mention that the phosphorylation of the β-catenin in the Ser552 by AKT may increase β-catenin/TCF-LEF activation possibly by association with histone acetylase [23]. In addition, coactivators recruited by β-catenin can determine which target genes are activated, and this differential recruitment can be regulated by phosphorylation [45]. Whether the complex ER/PI3K and the subsequent activation of AKT plays a role in expression and/or activation of β-catenin remains to be explored.

In conclusion, this study provides novel insights into molecular mechanisms of ER in androgen-independent prostate cancer cells. In PC-3 cells, ER activates rapid responses molecules, including SRC and PI3K/AKT. SRC is involved on the expression of the non-phosphorylated β-catenin. These events enhance the tumorigenic potential of prostate cancer cells PC-3, increasing cell proliferation, migration, invasion, and tumor formation. The complete mechanism by which ER are involved in CRPC is not fully understood, but it represents a promising new therapeutic avenue for advanced prostate cancer.

## Data Availability

The data presented in this study are available on request from the corresponding author.

## Supplementary Materials

The following are available online at https://www.mdpi.com/1422-0067/22/3/1153/s1, Figure S1: Effects of the selective inhibitor for SRC-family kinases (PP2) on the invasion of androgen-independent prostate cancer cells DU-145 induced by 17β-estradiol. Figure S2. Effects of DPN on non-phosphorylated β-catenin expression in androgen-independent prostate cancer cells PC-3.
