# Supplementary material for: Estrogen Receptor Signaling Pathways Involved in Invasion and Colony Formation of Androgen-Independent Prostate Cancer Cells PC-3
Source: Int J Mol Sci. 2021 Jan 25;22(3):1153. doi: 10.3390/ijms22031153 (PMC7865506; doi:10.3390/ijms22031153)
Supplement: Supplementary file 1 [file ijms-22-01153-s001.pdf]

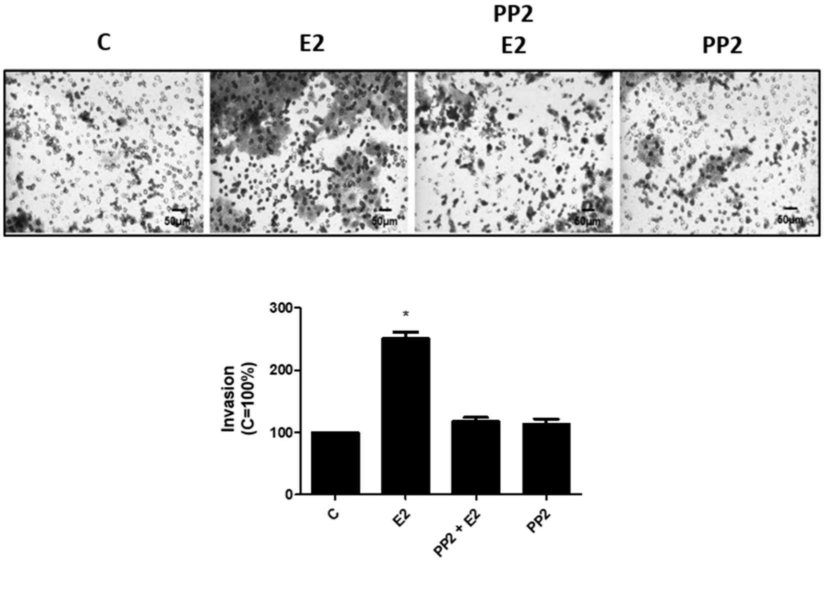

Figure S1: Effects of the selective inhibitor for SRC-family kinases (PP2) on the invasion of androgen-independent prostate cancer cells DU-145 induced by 17β-estradiol.

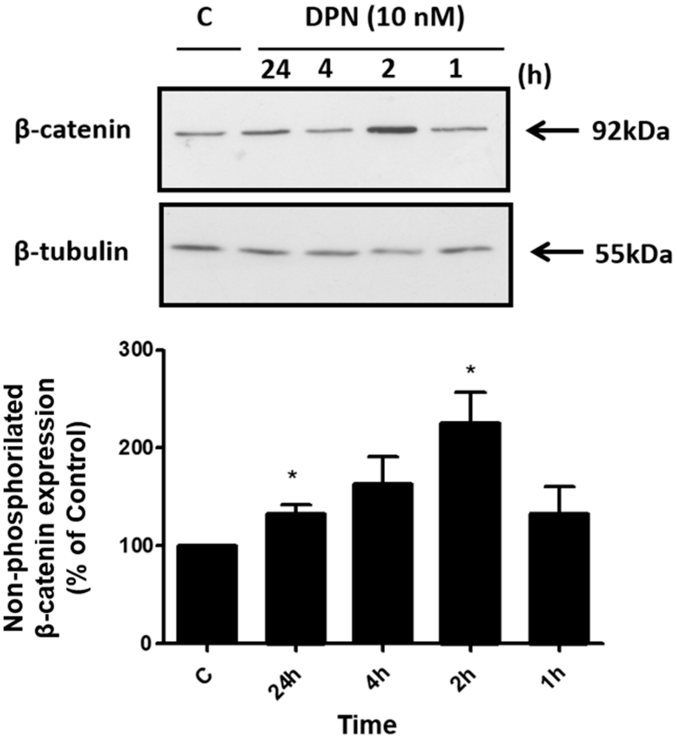

Figure S2. Effects of DPN on non-phosphorylated β-catenin expression in androgen-independent prostate cancer cells PC-3.
